# Combined pulse wave velocity and triglyceride–glucose index to discriminate large-artery atherosclerosis from small-vessel occlusion in acute ischaemic stroke: a single-centre retrospective study

**DOI:** 10.3389/fneur.2026.1775302

**Published:** 2026-03-31

**Authors:** Zhe Li, Ran Hui, Xiaoyun Guo, Qinmao Fang, Heping Deng

**Affiliations:** 1 Department of Ultrasound, Hebei Medical University Third Hospital, Shijiazhuang, Hebei, China; 2 Cerebral Blood Flow Graph Room, Hebei Medical University Third Hospital, Shijiazhuang, Hebei, China; 3 Department of Ultrasound, Hebei Medical University Second Hospital, Shijiazhuang, Hebei, China

**Keywords:** acute ischaemic stroke, large-artery atherosclerosis, predictive model, pulse wave velocity, small-vessel occlusion, triglyceride–glucose index

## Abstract

**Objective:**

Early etiologic discrimination between large-artery atherosclerosis (LAA) and small-vessel occlusion (SVO) is clinically relevant when advanced imaging is unavailable or delayed. We examined whether combining carotid pulse wave velocity (PWV) with the triglyceride–glucose (TyG) index improves early in - hospital discrimination of LAA vs. SVO, and developed a predictive model integrating PWV, TyG, and clinical indicators.

**Methods:**

We performed a single-centre retrospective study including consecutive acute ischaemic stroke patients from September 2021 to November 2023. Carotid PWV (Wv, m/s) was measured bilaterally and averaged; TyG was calculated from fasting triglycerides and glucose. Restricted cubic splines (RCS) tested non-linearity. Multivariable logistic regression was applied to (i) estimate the adjusted association of PWV and TyG with LAA versus SVO, and (ii) identify independent predictors via backward stepwise selection and establish predictive model. Discrimination was assessed by ROC AUC with DeLong tests; calibration used bootstrap-corrected curves; internal validation used 1,000-bootstrap optimism correction; decision-curve analysis appraised clinical utility.

**Results:**

Of 892 admissions screened, 298 were analysed (SVO 179; LAA 119). LAA showed higher PWV (median 17.72 vs. 15.02 m/s; *p* < 0.001) and higher TyG (8.85 vs. 8.64; *p* = 0.005) than SVO. RCS supported overall associations of PWV and TyG with LAA versus SVO without evidence of non-linearity. Compared with single markers (AUC: PWV = 0.627; TyG = 0.596), a two-marker model (PWV + TyG) achieved AUC 0.654, while a parsimonious four-variable model (PWV, TyG, sex, eGFR) reached AUC 0.685 and significantly outperformed either single marker (paired DeLong vs. PWV: ΔAUC 0.058, *p* = 0.043; vs. TyG: ΔAUC 0.089, *p* = 0.011). Calibration was acceptable after bootstrap correction.

**Conclusion:**

PWV and TyG provide complementary, early in - hospital signals for moderate discrimination of LAA vs. SVO, with incremental value when combined. The predictive model constructed by combining PWV, TyG, and clinical indicators also performed well. These bedside markers provide adjunctive, early in-hospital risk stratification for suspected LAA versus SVO.

## Introduction

Stroke remains a leading cause of death and disability worldwide, with its burden escalating despite advancements in prevention and treatment. Recent national estimates emphasize significant lifetime risk and increasing prevalence, underscoring the necessity for improved point-of-care etiological classification (1). Acute ischemic stroke (AIS) represents roughly 70% of cases, and is the predominant subtype (2). The two predominant subtypes within the Trial of Org 10,172 in Acute Stroke Therapy (TOAST) classification system of large-artery atherosclerosis (LAA) and small-vessel occlusion (SVO) exhibit distinct pathophysiological mechanisms, secondary prevention strategies, and prognostic characteristics (3). LAA is typically driven by atherosclerotic plaque burden with luminal stenosis or occlusion in large extracranial or intracranial arteries supplying the ischemic territory, whereas SVO is more often related to arteriolosclerosis with lipohyalinosis and microvascular endothelial injury affecting penetrating arterioles and producing small deep infarcts. However, in everyday practice, early subtyping often relies on advanced vascular imaging and magnetic resonance imaging (MRI), which are not always available upon admission (4); non-contrast computed tomography (CT) is not sensitive to small deep infarcts (5); clinical syndromes can overlap (6); and TOAST assignment usually follows a comprehensive evaluation completed after the hyperacute phase (3). Against this backdrop, having an etiological signal available early in the hospital to differentiate LAA from SVO becomes clinically crucial.

Recognizing the unmet need for etiologic cues available early in the hospital to distinguish LAA from SVO, integrating objective, non - invasive vascular measurements with readily obtainable serum markers to enable multidimensional discrimination early in the hospital has become a clinically relevant approach (7–9). Among these candidate tools, as a noninvasive metric of large-artery stiffness, pulse wave velocity (PWV) stands out (10). In particular, carotid PWV, which quantifies carotid artery stiffness, aids in early diagnosis and identification of vascular diseases such as carotid atherosclerosis (11). Higher PWV correlates with carotid atherosclerotic burden and has been associated with imaging markers of cerebral small-vessel disease, supporting its relevance across ischemic stroke phenotypes (12). While PWV cannot replace MRI for detecting small deep infarcts, it provides complementary, early in - hospital information that may enhance early etiologic discrimination when advanced imaging is unavailable or delayed (13, 14). Furthermore, serum markers that reflect endothelial dysfunction and atherosclerosis also demonstrate predictive value (7–9). The triglyceride-glucose (TyG) index, calculated from routinely available fasting triglyceride and glucose data within routine inpatient workflows, serves as a practical surrogate for insulin resistance (IR) (15). This index has been established to show clear epidemiological associations with macrovascular diseases (e.g., carotid atherosclerosis) and microvascular complications (16, 17). Emerging cerebrovascular studies further associate higher TyG with adverse features or outcomes after ischemic stroke (e.g., early lesion progression or early neurological deterioration), with signals that may be particularly relevant in atherosclerotic phenotypes (18). Despite these individual associations, it remains unknown whether combining a vascular stiffness marker (PWV) with a metabolic proxy (TyG) improves early in - hospital discrimination of LAA versus SVO beyond either marker alone. Prior population studies integrating TyG with estimated PWV address incident stroke risk, not etiologic subtyping within acute ischemic stroke (9). This evidence gap motivates the present, targeted investigation.

Above all, this study aimed to evaluate the discriminative value of PWV and the TyG index for early in - hospital discrimination of LAA from SVO and to quantify their incremental contribution beyond routine clinical covariates.

## Methods

### Study design and participants

This retrospective, single-centre cohort included consecutive acute cerebrovascular presentations (suspected stroke/TIA) admitted from September 2021 to November 2023. Inclusion criteria were: (1) age ≥18 years; (2) first-ever cerebrovascular event, defined as no documented history of symptomatic stroke or transient ischaemic attack in prior medical records and index admission history; (3) last-known-well to admission ≤24 h, determined from patient report or witness information recorded in the emergency or referral medical documentation; (4) neuroimaging with CT and/or MRI in accordance with contemporary guidelines (19); and (5) an aetiologic work-up sufficient for TOAST classification (3). Exclusion criteria were: (1) intracerebral haemorrhage; (2) transient ischaemic attack; (3) cerebral venous thrombosis; (4) cardioembolism or high-risk cardiac sources (20); (5) other determined aetiologies; (6) undetermined or multiple aetiologies after routine work-up; (7) invalid exposure measurement—defined as quality-control–failed or non-interpretable PWV recordings (21) and non-fasting or treatment-confounded lipid/glucose sampling (including: i A dextrose bolus or ongoing dextrose infusion within 2 h (22); ii An insulin bolus or infusion within 5 h systemically (22); iii Glucocorticoids within 24 h for intermediate-acting agents or within 36 h for dexamethasone (23); and iv Exposure to lipid injectable emulsions or propofol within 6 h before triglyceride sampling (24)) precluding valid TyG calculation; (8) severe hepatic dysfunction (Child–Pugh B/C) or advanced kidney disease (eGFR < 30 mL/min/1.73 m^2^), given their established associations with abnormal hemodynamics and arterial stiffness (25), and with altered insulin and lipid/glucose metabolism (14), potentially introducing non-etiology–related variation in PWV and TyG; (9) pregnancy or lactation; (10) incomplete or missing key data for candidate predictors required for modeling (Supplementary Table 1).

All the data were accessed for research purposes on September 1, 2025. The institutional ethics committee approved the study (approval number: W2020-040-1) and waived the requirement for informed consent owing to its retrospective design and de-identification of patient data. The study was conducted in accordance with the Declaration of Helsinki (26) and adhered to the Strengthening the Reporting of Observational Studies in Epidemiology (STROBE) guidelines (27).

### Data collection and measurements

Baseline demographics (age, gender, and admission body mass index [BMI]), vascular risk factors (smoking, hypertension, diabetes, duration of hypertension and diabetes [years], cardiovascular disease, previous stroke, renal disease, family history of cardiovascular disease, and left ventricular hypertrophy) were abstracted from the medical record. Fasting laboratory tests were obtained on the first morning after admission (≥8-h fast) and included fasting blood glucose (FBG), glycated haemoglobin (HbA1c), total cholesterol (TC), triglycerides (TG), HDL-C, LDL-C, serum creatinine (CREA), and apolipoprotein B (ApoB). The TyG index was calculated as ln[TG (mg/dL) × FBG (mg/dL) / 2] (28), with values in mmol/L converted beforehand (FBG × 18.02; TG × 88.57). Estimated glomerular filtration rate (eGFR; mL/min/1.73 m^2^) was derived using the CKD-EPI 2021 race-free equation from CREA (29).

Carotid local pulse wave velocity (local carotid PWV; device output Wv, in m/s) was defined as the wave propagation speed along a short common carotid segment and was interpreted as a segmental index of carotid arterial stiffness and compliance, with higher Wv indicating increased segmental stiffness and reduced compliance. This local carotid metric is conceptually distinct from guideline referenced carotid femoral PWV, which reflects global aortic stiffness, and therefore is not numerically interchangeable with cfPWV. Ultrasound based approaches for local PWV assessment have demonstrated good accuracy and reproducibility in prior work (30). Local carotid PWV was measured bilaterally at the level of the thyroid cartilage using a GT 3000 Cerebrovascular Function Detector (Shanghai Shenzhou Meijing Health Technology Co., Ltd., China) equipped with a 5 MHz Doppler probe and a pulsatile pressure sensor that synchronously acquire carotid flow and arterial elasticity related signals to compute Wv (11). The measurements were completed within 24 h of admission. Participants rested supine for at least 15 min before measurement, care was taken to avoid probe compression, and recordings were obtained near end expiration. Each side was recorded in triplicate and averaged, and the patient level PWV was defined *a priori* as the mean of left and right side values. Tracings with irregular rhythm or device quality control flags were repeated, and non interpretable signals were excluded per protocol. Examinations were performed by a trained sonographer blinded to laboratory data, in accordance with established CVHI quality control procedures and current recommendations for validation of noninvasive PWV devices (14, 31).

Aetiological subtypes were assigned according to the TOAST classification (3) and contemporary etiologic classification standards (32) using the complete index-admission etiologic work-up. All included patients underwent brain imaging (CT and/or MRI), vascular evaluation of both extracranial and intracranial arteries (carotid duplex ultrasound and/or computed tomography angiography/magnetic resonance angiography, with digital subtraction angiography when clinically indicated), and cardiac evaluation documented in the medical record. LAA was assigned when there was atherosclerotic stenosis ≥50% or occlusion of an extracranial or intracranial large artery relevant to the index infarct territory, together with no high-risk cardiac source of embolism (3, 20). SVO was assigned only when all of the following were met: a classic lacunar clinical syndrome without cortical signs, a compatible single small deep infarct on neuroimaging (≤15 mm in maximum diameter) in a perforator territory, no evidence of ≥50% stenosis or occlusion in the relevant parent large artery, and no high-risk cardiac sources or other determined etiology after the etiologic work-up (3, 20). Patients were grouped as LAA or SVO for the prespecified analysis. Two trained study personnel, blinded to PWV and the laboratory data used for TyG calculation, independently assigned TOAST subtype based on the prespecified criteria and the full work-up; discrepancies were resolved by consensus review.

### Candidate predictors and coding

The candidate predictor set comprised age, sex, BMI, hypertension (HT) and hypertension duration (HTY), diabetes (DM) and diabetes duration (DMY), history of stroke, smoking, cardiovascular disease, renal disease, family history, left ventricular hypertrophy, ApoB, HbA1c, HDL-C, LDL-C, total cholesterol, triglycerides, fasting blood glucose, creatinine, eGFR, carotid local PWV, and TyG. Sex was modeled as a binary factor with Female as the reference category. Continuous variables were entered as continuous terms as prespecified.

### Statistical analysis

Continuous variables were summarised as mean ± SD when approximately normal and as median (IQR) otherwise; categorical variables as counts (%). Between-group comparisons used Welch’s t test or the Wilcoxon rank-sum test for continuous variables and χ^2^ tests with Monte-Carlo simulation for categorical variables, as appropriate.

Linearity of the associations of PWV and TyG with LAA was evaluated using restricted cubic splines (RCS) with 4 knots placed at the 5th, 35th, 65th, and 95th percentiles of each predictor distribution. Overall and non-linear components were assessed using likelihood-ratio tests comparing models with and without spline terms. Given no evidence of non-linearity, both predictors entered subsequent models as continuous linear terms. A prespecified multivariable logistic-regression model adjusted for age, sex, smoking status, hypertension, ApoB, eGFR, and HbA1c was fitted to assess the independent associations of PWV and TyG. Backward elimination (Akaike Information Criterion) was performed starting from all variables with univariable *p* < 0.10 plus PWV and TyG (forced inclusion). The final four-variable model retained PWV, TyG, sex, and eGFR. Discrimination was quantified by ROC AUC for PWV alone, TyG alone, the two-marker model (PWV + TyG), and the four-variable model; AUCs and 95% CIs were obtained with the DeLong method, and paired DeLong tests compared each curve against the four-variable model on the same subjects. Sensitivity and specificity were reported at the Youden-optimal threshold.

Subgroup analyses were prespecified to evaluate the stability of discrimination of the four-variable model across clinically relevant strata, including age (<65 vs. ≥ 65 years), diabetes (yes vs. no), and hypertension (yes vs. no). For each subgroup, AUC and 95% CI were estimated using DeLong’s method based on the predicted probabilities from the prespecified four-variable model. AUCs were compared between subgroup strata using DeLong tests. In addition, we tested for effect modification by fitting logistic models including the model linear predictor, the subgroup indicator, and their interaction, and reported the likelihood-ratio test *p* value for the interaction term.

A prognostic nomogram was subsequently developed by integrating the four-variable model to estimate the individual LAA probability. Calibration was assessed using apparent and bootstrap bias-corrected calibration curves and was further quantified by calibration-in-the-large (intercept) and calibration slope. Optimism-corrected intercept and slope were obtained by bootstrap resampling with 1,000 iterations. Internal validation used bootstrap resampling (*B* = 1,000) to estimate optimism-corrected performance, reporting the optimism-corrected C-index and Brier score. Clinical utility was examined using decision-curve analysis (DCA) across prespecified threshold probabilities. To operationalize triage thresholds for the risk stratified nomogram, we selected two ROC derived probability cutpoints targeting sensitivity closest to 0.90 and specificity closest to 0.90, and mapped them to Total Points using the nomogram formula, presenting the corresponding low intermediate high strata.

Several sensitivity analyses were conducted to assess robustness. First, carotid local PWV was alternatively defined using the unilateral maximum value (maxPWV) instead of the prespecified bilateral mean, and the four - variable model was refitted. Second, we repeated the four - variable model after additionally including the 12 patients with severe hepatic dysfunction (Child - Pugh B/C) or advanced kidney disease (eGFR < 30 mL/min/1.73 m^2^) who were excluded in the primary analysis. Third, we performed multiple imputation by chained equations using an imputation model that included the outcome and all candidate predictors, and subsequently refitted and pooled the prespecified four-variable model across imputed datasets. Fourth, we assessed robustness to model specification by comparing a prespecified clinical model with and without PWV and TyG using DeLong tests, and by performing LASSO with 10-fold cross-validation (lambda.1se) followed by refitting the selected predictors and evaluating AUC.

### Events per variable

In the complete-case development dataset, there were 119 LAA events among 298 patients. Events-per-variable was 13.2 for the prespecified 9-predictor multivariable model (PWV, TyG, and 7 covariates) and 29.8 for the final four-variable model, supporting model parsimony and limiting overfitting.

### Missing data

Missing data were summarized for all candidate predictors and reported as counts and percentages. The primary analyses were performed as complete-case analyses after prespecified eligibility criteria were applied. We generated 20 imputed datasets and pooled estimates using Rubin’s rules. Because eGFR was derived from creatinine, age, and sex using the prespecified equation, eGFR was treated as a passive (derived) variable and recalculated within each imputed dataset rather than being imputed directly. Because missingness mechanisms cannot be proven in retrospective data, we assumed missing at random for the imputation model and acknowledged that missing not at random cannot be fully excluded.

All analyses were conducted using R software (version 4.3.2; R Foundation for Statistical Computing).

## Results

### Study population and baseline characteristics

**Figure 1:**
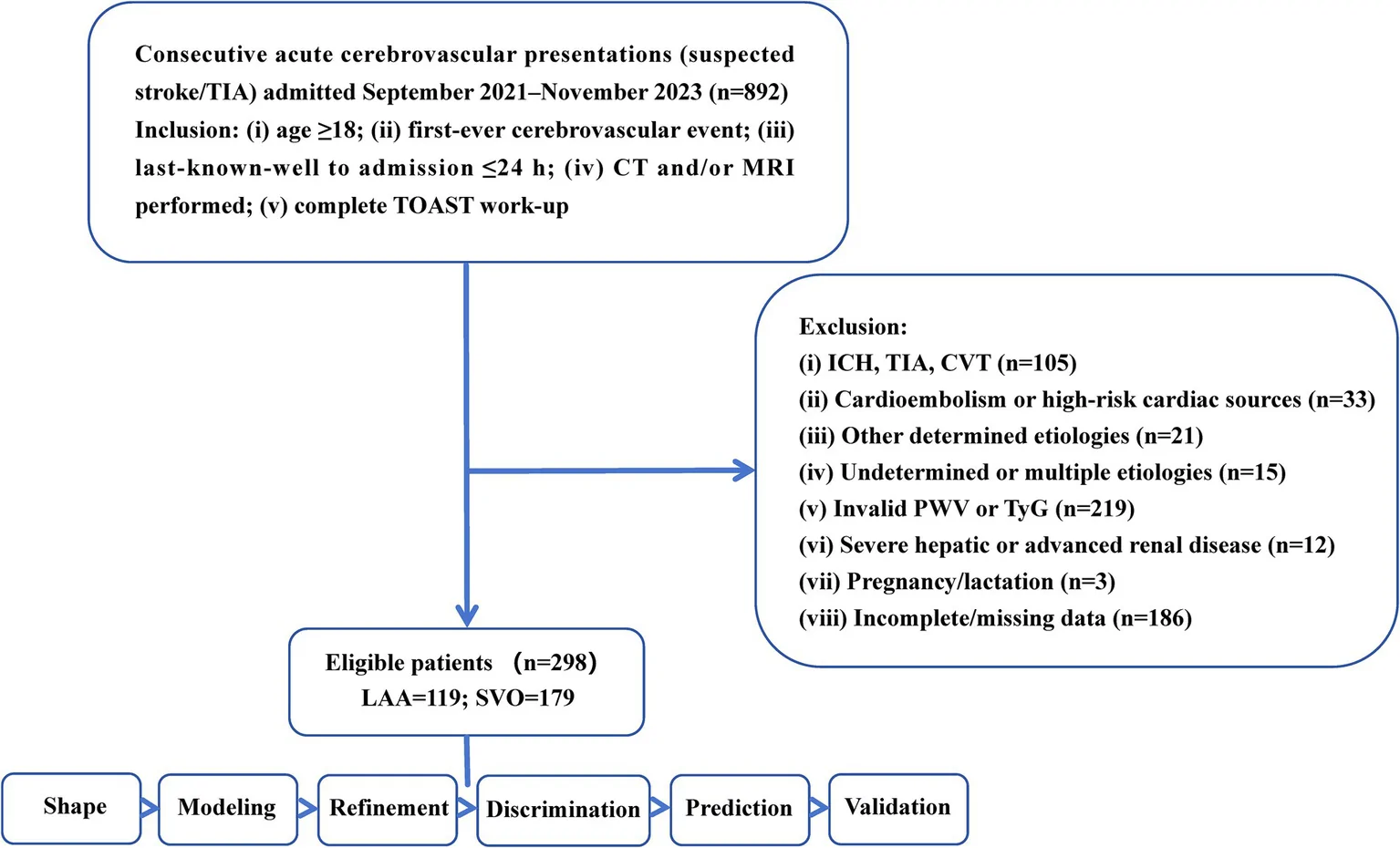
Flow diagram of patient selection and cohort allocation. TIA, Transient ischaemic attack; CT, computed tomography; MRI, magnetic resonance imaging; TOAST, Trial of ORG 10172 in acute stroke treatment classification; ICH, intracerebral haemorrhage; CVT, cerebral venous thrombosis; PWV, pulse wave velocity; TyG, triglyceride–glucose index; LAA, large-artery atherosclerosis; SVO, small-vessel occlusion.

Of the 892 screened admissions, a total of 298 patients were included: 179 with SVO and 119 with LAA. These 298 patients met the eligibility criteria and were analyzed; reasons for exclusion are detailed in Figure 1. The carotid local PWV measurement was performed at 5.0 [3.0, 8.0] hours post-admission, while the fasting laboratory tests for TyG calculation were obtained at 4.0 [2.5, 6.5] hours post-admission (Supplementary Figures 1A,B). Compared with SVO, the LAA group had higher PWV (median 17.72 vs. 15.02 m/s, *p* < 0.001), higher TyG (8.85 vs. 8.64, *p* = 0.005), higher FBG (6.10 vs. 5.80 mmol/L, *p* = 0.015), and lower HDL-C (1.08 vs. 1.18 mmol/L, *p* = 0.008). Hypertension was more frequent in LAA (69.7% vs. 57.5%, *p* = 0.032), and diabetes was also more frequent (43.7% vs. 30.2%, *p* = 0.023). Duration of diabetes was longer in LAA [median 0.00 (0.00–5.00) vs. 0.00 (0.00–1.00) years, *p* = 0.007]. Age did not significantly differ between groups (SVO 63.85 ± 11.55 years vs. LAA 66.00 ± 11.25 years, *p* = 0.112). Other lipids and kidney indexes showed no statistically significant differences (Table 1). Among screened admissions, 186 (20.9%) were excluded due to incomplete or missing data. Variable-level missingness for candidate predictors is summarized in Supplementary Table 1.

**Table 1:** Baseline characteristics of the study population.

Characteristic	Overall *N* = 298	SVO *N* = 179	LAA *N* = 119	*P*
Age (years)	64.71 ± 11.46	63.85 ± 11.55	66.00 ± 11.25	0.112
Gender				0.058
Female	118 (39.6%)	79 (44.1%)	39 (32.8%)	
Male	180 (60.4%)	100 (55.9%)	80 (67.2%)	
BMI (kg/m^2^)	24.55 [22.32, 26.84]	24.22 [22.15, 26.57]	24.77 [22.60, 27.20]	0.308
Hypertension	186 (62.4%)	103 (57.5%)	83 (69.7%)	0.032*
Hypertension years	2.00 [0.00, 8.00]	1.00 [0.00, 7.00]	5.00 [0.00, 9.00]	0.134
Diabetes	106 (35.6%)	54 (30.2%)	52 (43.7%)	0.023*
Diabetes years	0.00 [0.00, 2.00]	0.00 [0.00, 1.00]	0.00 [0.00, 5.00]	0.007*
History of stroke	69 (23.2%)	42 (23.5%)	27 (22.7%)	0.893
Smoking	67 (22.5%)	37 (20.7%)	30 (25.2%)	0.381
Cardiovascular disease	57 (19.1%)	32 (17.9%)	25 (21.0%)	0.548
Renal disease	18 (6.0%)	11 (6.1%)	7 (5.9%)	1.000
Family history	46 (15.4%)	24 (13.4%)	22 (18.5%)	0.243
Left ventricular hypertrophy	12 (4.0%)	7 (3.9%)	5 (4.2%)	1.000
TG (mmol/L)	1.22 [0.91, 1.69]	1.18 [0.87, 1.66]	1.27 [1.01, 1.78]	0.063
FBG (mmol/L)	5.90 [5.10, 7.80]	5.80 [4.84, 7.20]	6.10 [5.20, 8.10]	0.015*
HbA1c (%)	6.10 [5.60, 7.50]	6.00 [5.60, 7.40]	6.10 [5.60, 7.60]	0.990
LDL-C (mmol/L)	2.52 [1.92, 3.16]	2.49 [1.92, 3.11]	2.60 [1.90, 3.27]	0.337
HDL-C (mmol/L)	1.12 [0.98, 1.32]	1.18 [0.98, 1.35]	1.08 [0.96, 1.23]	0.008*
TC (mmol/L)	4.28 ± 1.24	4.33 ± 1.18	4.21 ± 1.33	0.443
ApoB (g/L)	0.88 [0.70, 1.06]	0.86 [0.70, 1.03]	0.91 [0.71, 1.10]	0.146
CREA (umol/L)	68.53 [57.00, 79.49]	67.00 [57.00, 78.48]	71.81 [57.00, 81.96]	0.110
eGFR (mL/min/1.73 m^2^)	95.51 [85.07, 102.59]	96.46 [85.77, 103.35]	94.29 [83.94, 100.96]	0.091
TyG	8.72 [8.29, 9.17]	8.64 [8.23, 9.10]	8.85 [8.42, 9.28]	0.005*
PWV (m/s)°	16.11 [12.62, 21.33]	15.02 [11.84, 19.56]	17.72 [14.20, 23.24]	<0.001*

*n* (%); Mean ± SD; Median [Q1, Q3]; Pearson’s Chi-squared test with simulated *p*-value (based on 2000 replicates); Welch Two Sample *t*-test; Wilcoxon rank sum test; °Carotid PWV reference value was defined as the mean of left and right sides, with a normal cut-off of ≤21.56 m/s (derived from the normal reference values of left ≤21.00 m/s and right ≤22.12 m/s, as specified in cerebrovascular function test reports); *p < 0.05. Abbreviations: SVO, small-vessel occlusion; LAA, large-artery atherosclerosis; BMI, body mass index; TG, triglycerides; FBG, fasting blood glucose; HbA1c, glycated haemoglobin; LDL-C, low-density lipoprotein cholesterol; HDL-C, high-density lipoprotein cholesterol; TC, total cholesterol; ApoB, apolipoprotein B; CREA, serum creatinine; eGFR, estimated glomerular filtration rate; TyG, triglyceride–glucose index; PWV, pulse wave velocity.

### Linearity assessment (RCS)

**Figure 2:**
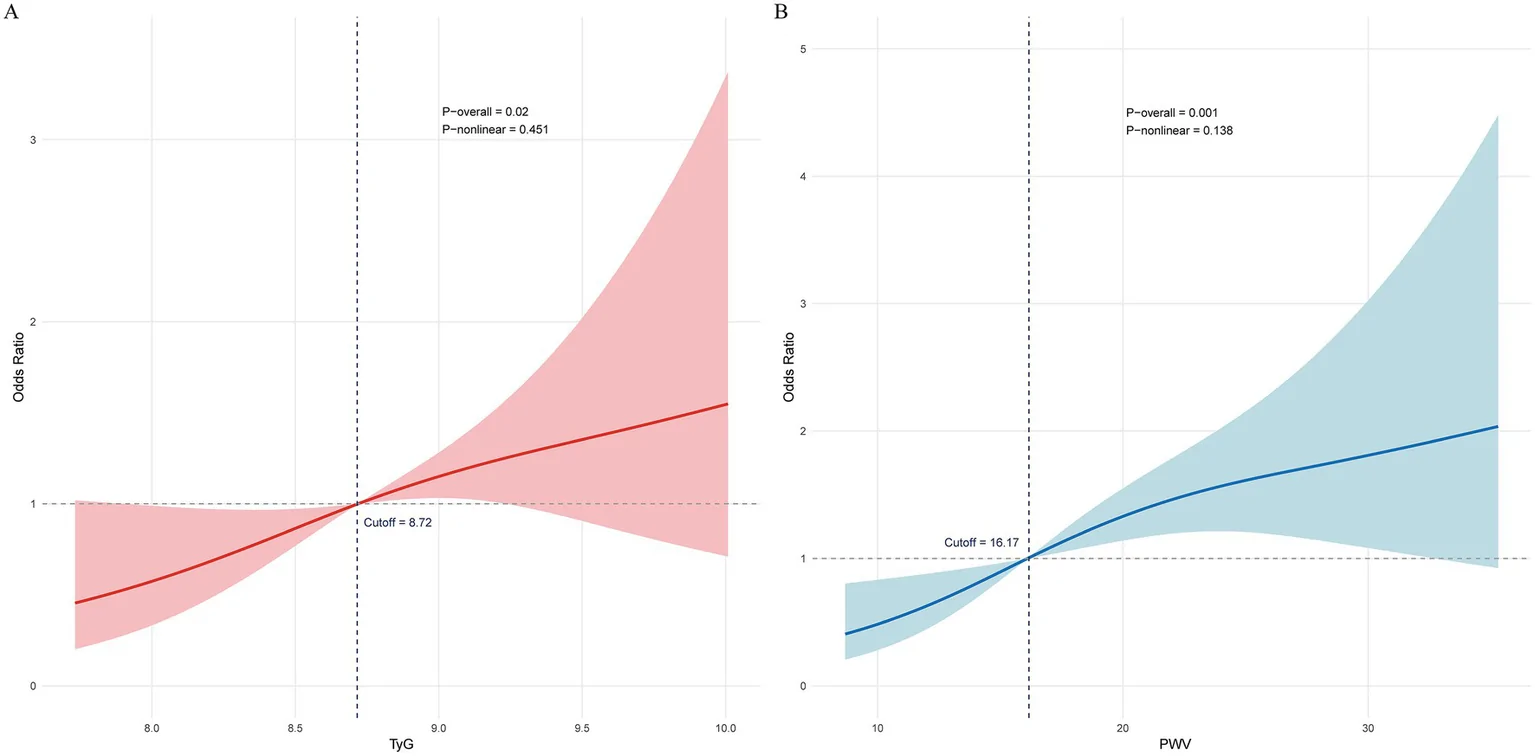
Restricted cubic spline associations of pulse wave velocity (PWV, **A**) and triglyceride–glucose (TyG, **B**) index with the odds of large-artery atherosclerosis (LAA) compared with small-vessel occlusion (SVO), using 4 knots placed at the 5th, 35th, 65th, and 95th percentiles. The curves are presented for assessment of functional form and risk gradients, and no clinical cutoff was derived from any visual turning point.

Spline analyses supported overall associations of TyG and PWV with the odds of LAA versus SVO without evidence of non-linearity (TyG: *P*-overall = 0.020, *P*-nonlinear = 0.451; PWV: *P*-overall = 0.001, *P*-nonlinear = 0.138) (Figure 2). Accordingly, PWV and TyG were modeled as continuous linear predictors in subsequent analyses.

### Multivariable associations

In the prespecified multivariable model, both haemodynamic and metabolic markers remained independently associated with LAA (vs SVO): PWV (OR 1.06 per 1 m/s, 95% CI 1.02–1.10, *p* = 0.004) and TyG (OR 1.79 per unit, 95% CI 1.13–2.85, *p* = 0.014); male sex was also associated with LAA (OR 2.15, 95% CI 1.21–3.84, *p* = 0.009), whereas age, smoking, hypertension, ApoB, eGFR, and HbA1c were not statistically significant after mutual adjustment (Table 2).

**Table 2:** Multivariable logistic regression for LAA versus SVO*.

Characteristic	OR	95%CI	*P*
PWV(m/s)°	1.06	1.02–1.10	0.004
TyG	1.79	1.13–2.85	0.014
Age (years)	1.01	0.98–1.03	0.534
Gender (Male)	2.15	1.21–3.84	0.009
Smoking	1.18	0.62–2.24	0.617
Hypertension	1.19	0.68–2.08	0.538
ApoB (g/L)	1.71	0.66–4.41	0.266
eGFR (mL/min/1.73 m^2^)	0.99	0.98–1.00	0.101
HbA1c (%)	0.91	0.77–1.07	0.240

*Model adjusted for age (years), sex (male vs. female), smoking status, hypertension (yes/no), ApoB, eGFR (mL/min/1.73 m^2^), and HbA1c (%); PWV (m/s) and TyG were the exposures of interest.

Carotid PWV reference value was defined as the mean of left and right sides, with a normal cut-off of ≤21.56 m/s (derived from the normal reference values of left ≤21.00 m/s and right ≤22.12 m/s, as specified in cerebrovascular function test reports).

LAA, large-artery atherosclerosis; SVO, small-vessel occlusion; PWV, pulse wave velocity; TyG, triglyceride–glucose index; ApoB, apolipoprotein B; eGFR, estimated glomerular filtration rate; HbA1c, glycated haemoglobin; OR, odds ratio; CI, confidence interval.

A parsimonious model derived by backward elimination retained four predictors—PWV, TyG, sex, and eGFR—with consistent effect sizes: PWV (OR 1.06 per 1 m/s, 95% CI 1.03–1.10, *p* < 0.001), TyG (OR 1.80 per unit, 95% CI 1.21–2.68, *p* = 0.004), male sex (OR 2.13, 95% CI 1.26–3.59, *p* = 0.005), and eGFR (OR 0.99 per 1 mL/min/1.73 m^2^, 95% CI 0.97–1.00, *p* = 0.037, Table 3).

**Table 3:** Final parsimonious model for discriminating LAA versus SVO.

Characteristic	OR	95%CI	*P*
PWV(m/s)°	1.06	1.03–1.10	<0.001
TyG	1.80	1.21–2.68	0.004
Gender (Male)	2.13	1.26–3.59	0.005
eGFR (mL/min/1.73 m^2^)	0.99	0.97–1.00	0.037

Carotid PWV reference value was defined as the mean of left and right sides, with a normal cut-off of ≤21.56 m/s (derived from the normal reference values of left ≤21.00 m/s and right ≤22.12 m/s, as specified in cerebrovascular function test reports).

LAA, large-artery atherosclerosis; SVO, small-vessel occlusion; PWV, pulse wave velocity; TyG, triglyceride–glucose index; eGFR, estimated glomerular filtration rate; OR, odds ratio; CI, confidence interval.

### Discrimination, incremental value, calibration, and internal validation

**Figure 3:**
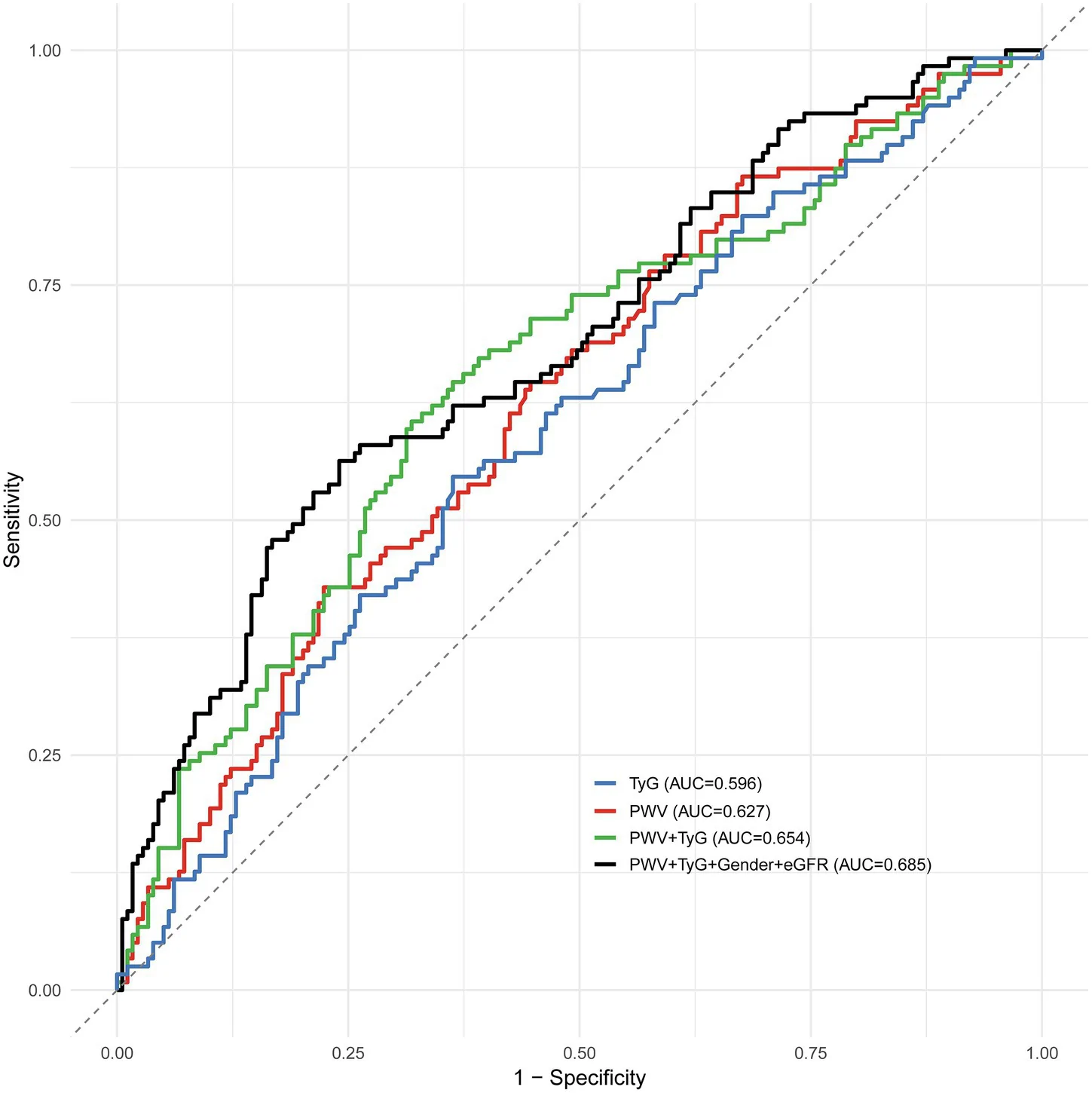
ROC curves comparing models for large-artery atherosclerosis (LAA) vs. small-vessel occlusion (SVO). PWV, pulse wave velocity; TyG, triglyceride-glucose; eGFR, estimated glomerular filtration rate.

Single-marker AUCs were 0.627 for PWV (95% CI 0.563–0.691) and 0.596 for TyG (95% CI 0.531–0.661). The two-variable model (PWV + TyG) achieved AUC 0.654 (95% CI 0.590–0.718), and the four-variable model (PWV + TyG + sex+eGFR) achieved AUC 0.685 (95% CI 0.623–0.747, Figure 3). In paired DeLong tests versus the four-variable model, discrimination improved significantly over PWV alone (ΔAUC = 0.058; *p* = 0.043) and TyG alone (ΔAUC = 0.089; *p* = 0.011), while the increment over PWV + TyG was modest and not statistically significant (ΔAUC = 0.031; *p* = 0.194). At the Youden-optimal threshold, the four-variable model yielded sensitivity 0.563 and specificity 0.760 (Table **4**).

**Table 4:** ROC performance and incremental discrimination relative to the four-variable model.

Variables	AUC	95% CI	Sensitivity	Specificity	Youden index	ΔAUC (4-var. − model)	*P* (paired DeLong vs 4-var)	*P* (AUC>0.5)
Four-variable model	0.685	0.623–0.747	0.563	0.760	0.323	Ref	—	<0.001
PWV° + TyG	0.654	0.590–0.718	0.605	0.682	0.287	0.031	0.194	<0.001
PWV°	0.627	0.563–0.691	0.429	0.777	0.205	0.058	0.043	<0.001
TyG	0.596	0.531–0.661	0.546	0.637	0.183	0.089	0.011	0.004

Outcome coded LAA = 1 and SVO = 0 (TOAST); ROC direction set to classify LAA as the positive class. For single predictors (PWV, TyG), AUCs are based on the continuous measurements; for combined models, AUCs use logistic-regression predicted probabilities. AUCs and 95% CIs were obtained by the DeLong method. ΔAUC (4-var. − model) is the absolute AUC difference comparing each predictor/model with the four-variable model on the same subjects; P (paired DeLong vs 4-var) is the corresponding paired DeLong test. *P* (AUC > 0.5) tests whether the AUC differs from 0.5. Sensitivity and specificity are reported at the Youden-optimal threshold for each curve; Youden’s J = Sensitivity + Specificity − 1.

Carotid PWV reference value was defined as the mean of left and right sides, with a normal cut-off of ≤21.56 m/s (derived from the normal reference values of left ≤21.00 m/s and right ≤22.12 m/s, as specified in cerebrovascular function test reports).

LAA, large-artery atherosclerosis; SVO, small-vessel occlusion; PWV, pulse wave velocity; TyG, triglyceride–glucose index; eGFR, estimated glomerular filtration rate; AUC, area under the ROC curve; CI, confidence interval.

Subgroup analyses showed broadly consistent discrimination of the four-variable model across age, diabetes, and hypertension strata (Table 5). AUCs were 0.683 (95% CI 0.595–0.772) in patients aged <65 years and 0.680 (0.593–0.767) in those aged ≥65 years (DeLong *p* = 0.962; interaction *p* = 0.636). In patients without versus with diabetes, AUCs were 0.667 (0.584–0.749) and 0.654 (0.550–0.758), respectively (DeLong *p* = 0.856; interaction *p* = 0.645). In patients without versus with hypertension, AUCs were 0.640 (0.533–0.747) and 0.677 (0.599–0.755), respectively (DeLong *p* = 0.588; interaction *p* = 0.994).

**Table 5:** Subgroup analyses of discrimination for the prespecified four-variable model.

Subgroup	Stratum	*N* (events)	AUC (95% CI)	*P* for AUC difference (DeLong)	*P* for interaction
Overall	All	298 (119)	0.674 (0.611–0.736)		
Age	<65	141 (57)	0.683 (0.595–0.772)	0.962	0.636
Age	≥65	157 (62)	0.680 (0.593–0.767)	0.962	0.636
Diabetes	No	192 (67)	0.667 (0.584–0.749)	0.856	0.645
Diabetes	Yes	106 (52)	0.654 (0.550–0.758)	0.856	0.645
Hypertension	No	112 (36)	0.640 (0.533–0.747)	0.588	0.994
Hypertension	Yes	186 (83)	0.677 (0.599–0.755)	0.588	0.994

AUC and 95% CI were estimated using DeLong’s method based on predicted probabilities from the prespecified four-variable model. *P* for AUC difference compares AUCs between strata using DeLong tests. *P* for interaction is from likelihood-ratio tests for the interaction between the model linear predictor and the subgroup indicator.

**Figure 4:**
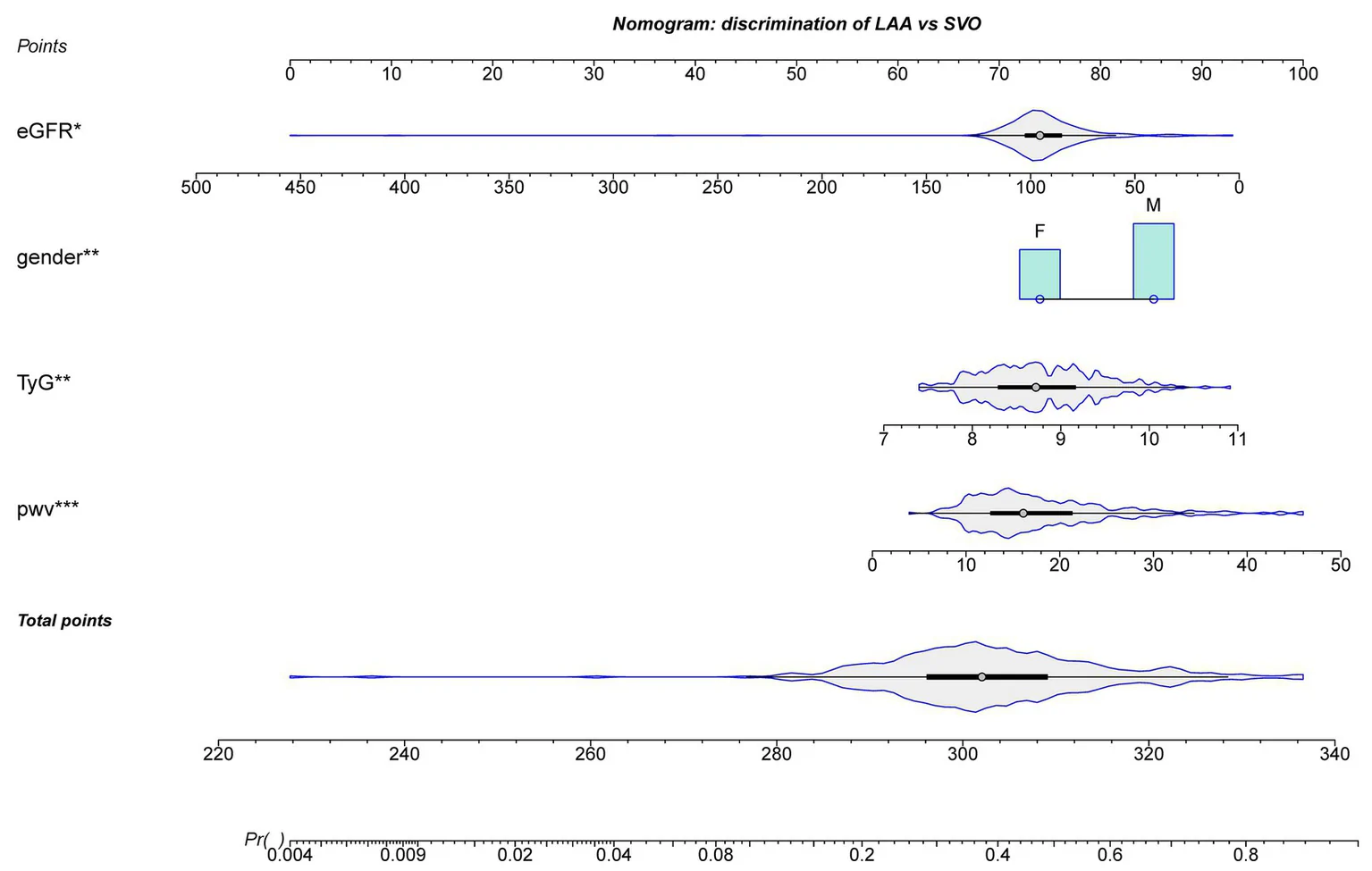
Nomogram for discriminating large-artery atherosclerosis (LAA) from small-vessel occlusion (SVO) using a four-variable model. PWV, pulse wave velocity; TyG, triglyceride-glucose; eGFR, estimated glomerular filtration rate.

**Figure 5:**
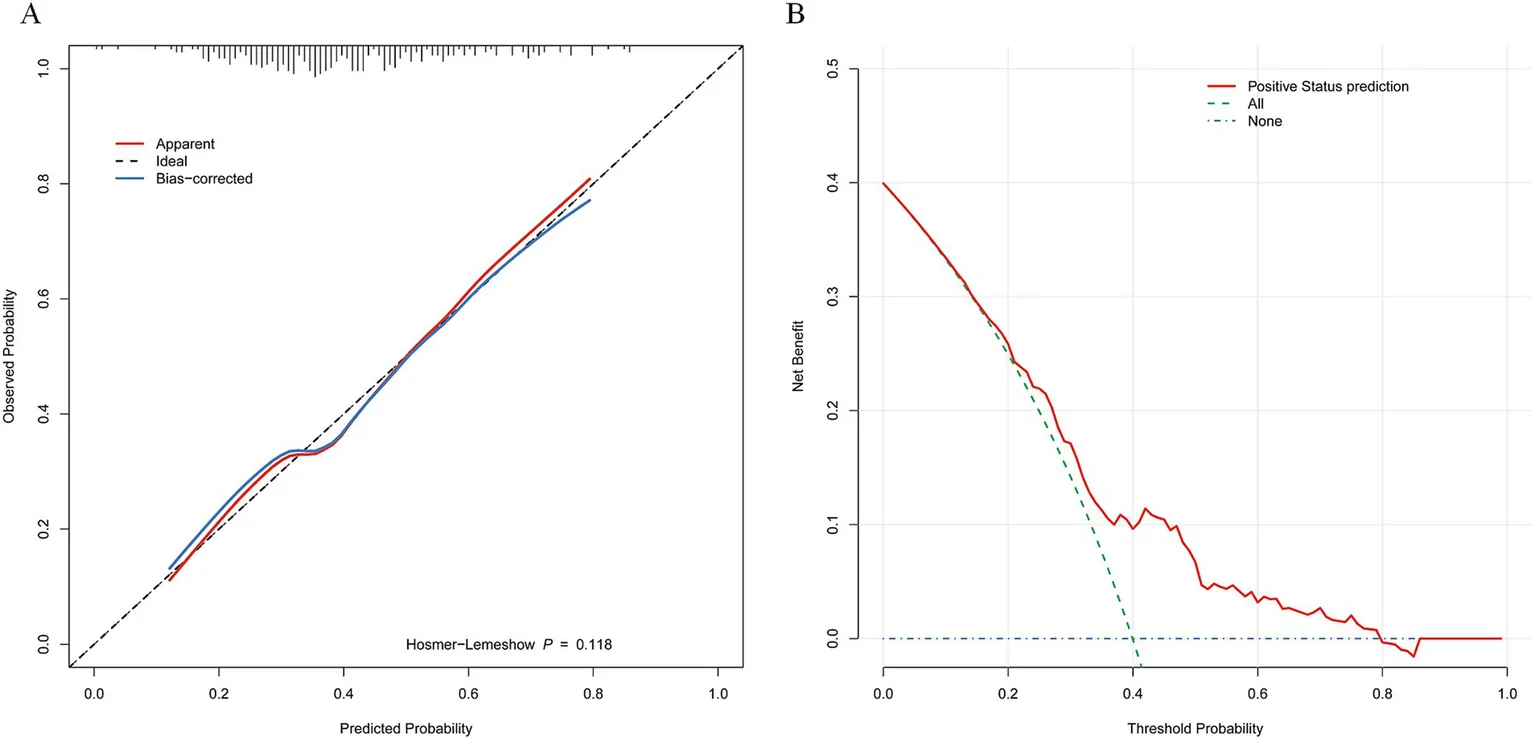
Calibration **(A)** and decision-curve analysis **(B)** of the four-variable model.

The final four-variable logistic model was *logit P(LAA) = −6.3871 + 0.06117 × PWV + 0.60633 × TyG + 0.75759 × Male - 0.01002 × eGFR* with Female as the reference category for sex. A nomogram incorporating these four predictors was constructed to facilitate bedside estimation of individual LAA probability; point allocations and the total-points scale are provided in Figure 4. The concordance index (C-index) and Brier score were 0.685 and 0.213, respectively. After internal validation using bootstrap resampling (1,000 iterations), the optimism-corrected C-index was 0.671 and the Brier score was 0.219, indicating good model stability and predictive performance. Calibration was good by visual inspection, with the apparent and bootstrap bias-corrected curves closely following the 45° line (Figure 5A). Quantitatively, the bootstrap optimism-corrected calibration intercept was −0.036 and the calibration slope was 0.908, indicating good overall calibration with mild overfitting. DCA demonstrated that the nomogram provided a positive net clinical benefit across a threshold probability range of 8 to 80% (Figure 5B). For threshold operationalization, the two probability cutpoints were 0.2673 and 0.5437, corresponding to Total Points cutpoints of 110.7 and 127.8 (Supplementary Figure 2).

### Sensitivity analysis

When PWV was alternatively aggregated as the unilateral maximum value (maxPWV), all four predictors remained statistically significant with similar effect sizes; When the 12 patients with severe hepatic or renal dysfunction were added back into the cohort, PWV, TyG, and sex remained significant with concordant directions, the eGFR association was attenuated and became borderline. In the multiple-imputation sensitivity analysis, PWV, TyG, and male sex remained significant, whereas the eGFR association was attenuated and no longer statistically significant. The prespecified clinical model achieved an AUC of 0.636, which increased to 0.690 after adding PWV and TyG (DeLong *p* = 0.0199). In the penalized-regression analysis, LASSO at lambda.1se selected DMY, HDL-C, PWV, and TyG; the refitted model yielded an AUC of 0.685 (Supplementary Tables 2–5).

## Discussion

The etiological classification of AIS directly influences subsequent treatment pathways and secondary prevention strategies. According to the TOAST criteria, LAA and SVO represent the most common subtypes, differing substantially in pathophysiological mechanisms, therapeutic intensity, and long-term prognosis (3). In real-world emergency settings, early subtyping often depends on vascular imaging and MRI, which may not be immediately accessible upon admission; non-contrast CT lacks sensitivity for small deep infarcts, clinical syndromes may overlap, and TOAST assignment typically occurs post-hyperacute phase. Therefore, early in - hospital bedside clues that suggest LAA or SVO likelihood hold clear clinical value without delaying reperfusion decisions.

The principal findings of this study indicate that baseline levels of PWV and the TyG index are elevated in LAA compared to SVO patients, with restricted cubic spline analyses supporting overall associations without evident nonlinearity. Multivariable modeling confirmed PWV, TyG, sex, and eGFR as independent predictors of LAA. Discriminative performance improved progressively from single markers to the combined PWV + TyG model and further to the four-variable model, with internal validation demonstrating good calibration and clinical utility across relevant threshold probabilities. These results suggest that integrating vascular stiffness (PWV) and metabolic burden (TyG) provides complementary early in - hospital information for moderate-strength differentiation of LAA from SVO.

Mechanistically, the TyG index, derived from fasting triglycerides and glucose, serves as a practical surrogate for IR, validated against clamp techniques and homeostatic model assessment (28). IR promotes atherosclerosis and plaque instability via lipid dysregulation, endothelial dysfunction, and inflammatory/oxidative stress pathways (33). Population and clinical studies have linked higher TyG to carotid atherosclerosis, ischemic stroke risk, and adverse outcomes, including early lesion progression and recurrence, particularly in atherosclerotic phenotypes (16, 18). PWV, a measure of arterial stiffness, is recommended in guidelines for assessing central artery elasticity, with carotid-femoral PWV as the reference standard (10). This study employed ultrasound/pressure-wave synchronized local carotid PWV (device output Wv, m/s), characterizing segmental large-artery compliance, distinct from brachial-ankle or estimated PWV metrics (11). As a local carotid measure, Wv primarily captures site specific arterial wall properties and the local hemodynamic milieu relevant to carotid atherosclerotic burden. Accordingly, Wv values and thresholds should not be interpreted using cfPWV cutoffs, and they should not be assumed to be numerically interchangeable with cfPWV or other PWV modalities across platforms. Arterial stiffness correlates with carotid atherosclerotic burden and cerebral small-vessel disease markers (e.g., white matter hyperintensities, lacunes), underpinning its relevance across ischemic phenotypes (34, 35). The independent associations of PWV and TyG observed herein align with their complementary roles—structural/functional stiffness versus metabolic/IR load—explaining the incremental discrimination upon combination, as echoed in recent studies integrating TyG with estimated PWV for stroke risk stratification (7, 9).

Clinically, PWV is not a substitute for MRI or vascular imaging in detecting small deep infarcts but may provide complementary clues during imaging delays (36, 37). These bedside-accessible markers, such as PWV and TyG, can guide imaging prioritization, phenotype-specific secondary prevention (e.g., intensified anti-atherosclerosis therapy for suspected LAA versus optimized small-vessel risk control for SVO), and resource allocation in patients eligible for reperfusion therapy (12, 38–40). Restricted cubic spline analyses suggested monotonic risk gradients of PWV and TyG for LAA versus SVO without evidence of non-linearity. Importantly, these spline curves were used to assess functional form and visualize risk gradients rather than to derive clinical thresholds. Future multicenter prospective studies should validate model generalizability across devices and populations, integrating with clinical/imaging scores to quantify benefits in dual antiplatelet therapy selection (41), imaging triage, or 3-month recurrence reduction via randomized designs. Given the modest AUC and sensitivity, the model is not intended to stand alone for etiologic assignment. Instead, it may serve as an adjunctive triage tool when definitive vascular imaging is delayed or constrained. We operationalized this concept using two ROC derived probability cutpoints for predicted LAA risk, reflecting common clinical trade offs between minimizing missed LAA and avoiding unnecessary expedited imaging. A lower cutpoint was selected to target sensitivity closest to 0.90, supporting a rule out triage threshold, whereas a higher cutpoint was selected to target specificity closest to 0.90, supporting a rule in prioritization threshold. In our cohort, these probability cutpoints were 0.2673 and 0.5437, respectively, which corresponded to Total Points cutpoints of 110.7 and 127.8 on the nomogram. Accordingly, patients with Total Points below 110.7 could undergo routine etiologic work up with greater emphasis on small vessel risk factor optimization, whereas patients with Total Points at or above 127.8 could be prioritized for expedited extracranial and intracranial vascular imaging and early emphasis on anti atherosclerotic secondary prevention. Intermediate risk, defined as Total Points 110.7 to 127.8, may be treated as indeterminate, prompting standard imaging and reassessment. The choice of thresholds should ultimately reflect local resource constraints and the relative consequences of missed LAA versus unnecessary expedited imaging, consistent with our decision curve analysis.

Although both carotid local PWV and the TyG index can be obtained early in hospital, these measures are physiologically dynamic and may be influenced by early in hospital interventions and acute stress (42–44). Local carotid PWV is pressure dependent (42) and sensitive to short term changes in hemodynamics and vascular tone, therefore intravenous fluids, blood pressure lowering, analgesia or sedation, and heightened sympathetic activation during the acute phase could shift arterial operating pressure and smooth muscle tone and lead to functional fluctuations in PWV beyond chronic structural stiffening (43, 44). In addition, acute inflammatory states may alter hemodynamics and can be accompanied by higher large artery stiffness, although reported effects vary by stimulus and population, which may further contribute to early PWV variability (45, 46). TyG is calculated from fasting triglycerides and fasting glucose (47), of which fasting glucose is particularly susceptible to stress hyperglycemia and early glucose management (48), while triglycerides are generally less labile but may still be affected by fasting status (49), hemodilution, and acute phase responses in acute stroke (50). Importantly, these early in hospital perturbations are more likely to introduce non differential measurement variability across etiologic subtypes, which would tend to attenuate associations toward the null and render our estimates conservative rather than spuriously inflating discrimination. Taken together, our early in hospital measurements likely reflect a composite of chronic vascular metabolic burden and acute physiologic perturbations, and future prospective studies with standardized timing and concurrent hemodynamic recording will be valuable to further improve transportability.

Medication related confounding cannot be fully excluded in the acute stroke setting. Chronic pre admission use and early in hospital initiation or intensification of lipid lowering and antihypertensive therapies can modify arterial stiffness and PWV, while glucose lowering therapy and acute glucose management can alter fasting glucose and thereby TyG (51). Patients with LAA may be more likely to receive intensive preventive regimens, which could lower TyG and reduce PWV and thus bias subtype differences toward the null, consistent with potential reverse causation. Stress hyperglycemia is common after acute ischemic stroke even in individuals without diabetes, and PWV is intrinsically blood pressure dependent, so short term hemodynamic shifts and treatment related blood pressure changes may contribute additional variability beyond chronic vascular metabolic burden (52). Accordingly, incomplete medication ascertainment and acute physiological stress may contribute to residual confounding, and prospective cohorts with standardized medication capture and concurrent hemodynamic recording would further improve transportability.

The strengths of this study encompass consecutive enrollment, rigorous predefined analytics with bootstrap validation to mitigate overfitting, and standardized PWV measurement. These enable robust discrimination between LAA and SVO using early in - hospital TyG and PWV, despite a modest sample size. Notably, sensitivity analyses supported the robustness of our conclusions. Redefining PWV using the unilateral maximum value did not materially alter effect estimates, and adding back the 12 severe hepatic or renal dysfunction cases preserved the overall directions while modestly attenuating the eGFR effect. This is consistent with the more extreme stiffness and metabolic profiles observed in this subgroup. In prespecified subgroup analyses, discrimination of the four-variable model remained broadly similar across age, diabetes, and hypertension strata, with overlapping confidence intervals and no statistical evidence of effect modification. This supports the stability of the model’s discriminative performance in clinically relevant subgroups. Both a prespecified clinical-model augmentation analysis and an alternative penalized-regression approach yielded consistent discrimination patterns and retained PWV and TyG as informative predictors, supporting that our main findings are not solely driven by stepwise variable selection.

Nevertheless, several limitations must be acknowledged. Firstly, the single-center, retrospective design and relatively modest sample size (*n* = 298) raise concerns about generalizability; therefore, the results need to be validated in a multi-center, large-sample setting. Secondly, although the instruments and corresponding carotid PWV assessment methods used at our institution have been applied in multicenter settings (53, 54), device derived local carotid Wv is inherently dependent on platform specific hardware and algorithms. Therefore, absolute values, reference ranges, and probability cutpoints may vary across devices and centers, and model coefficients and thresholds should not be assumed to transport directly to other platforms or to cfPWV measurements without external validation and recalibration. Multicenter external validation, ideally including head to head acquisition of guideline referenced cfPWV in a subset for calibration and harmonization, remains necessary. Third, residual confounding may persist, particularly from pre-admission medications that could influence admission lipid/glucose profiles or hemodynamic parameters. Fourth, the *a priori* focus on differentiating LAA from SVO necessarily excluded other TOAST categories, which narrows the case mix compared with unselected AIS populations and may introduce spectrum bias; therefore, model discrimination may differ when applied to cohorts including cardioembolism or undetermined/multiple etiologies. Fifth, although PWV and TyG were obtained early in hospital and we reported the timing distributions, detailed early in hospital management at the time of measurement such as fluid administration and glucose or blood pressure lowering therapy was not systematically captured. Therefore, residual confounding from acute management and stress responses cannot be excluded. Sixth, although we performed multiple imputation to assess robustness, the missingness mechanism cannot be definitively verified in this retrospective setting; therefore, some degree of residual selection bias due to incomplete data cannot be fully excluded.

## Conclusion

PWV and TyG provide complementary, early in - hospital signals for moderate discrimination of LAA vs. SVO, with incremental value when combined. The predictive model constructed by combining PWV, TyG, and clinical indicators also performed well. These bedside markers provide adjunctive, early in-hospital risk stratification for suspected LAA versus SVO.

## Data Availability

The raw data supporting the conclusions of this article will be made available by the authors, without undue reservation.
