# Correction: Combined pulse wave velocity and triglyceride–glucose index to discriminate large-artery atherosclerosis from small-vessel occlusion in acute ischaemic stroke: a single-centre retrospective study

**DOI:** 10.3389/fneur.2026.1994863

**Published:** 2026-09-23

**Authors:** Zhe Li, Ran Hui, Xiaoyun Guo, Qinmao Fang, Heping Deng

**Affiliations:** 1 Department of Ultrasound, Hebei Medical University Third Hospital, Shijiazhuang, Hebei, China; 2 Cerebral Blood Flow Graph Room, Hebei Medical University Third Hospital, Shijiazhuang, Hebei, China; 3 Department of Ultrasound, Hebei Medical University Second Hospital, Shijiazhuang, Hebei, China

**Keywords:** acute ischaemic stroke, large-artery atherosclerosis, predictive model, pulse wave velocity, small-vessel occlusion, triglyceride–glucose index

An incorrect **Funding** statement was erroneously published. The project associated with No. 20201037 was incorrectly identified as a “National Health Commission project.” The project number itself was reported correctly. The correct **Funding** statement appears below:

The author(s) declared that financial support was received for this work and/or its publication. This work was conducted under the framework of the Hebei Medical Science Research Project (No. 20201037).

The original version of this article has been updated.

