# Supplementary material for: Combined pulse wave velocity and triglyceride–glucose index to discriminate large-artery atherosclerosis from small-vessel occlusion in acute ischaemic stroke: a single-centre retrospective study
Source: Front Neurol. 2026 Mar 31;17:1775302. doi: 10.3389/fneur.2026.1775302 (PMC13076111; doi:10.3389/fneur.2026.1775302)
Supplement: Supplementary file 1 [file Data_Sheet_1.docx]

**Supplementary**

**Supplementary Table 1** Missingness of Candidate Predictors

| **Candidate predictor** | **Missing (n)** | **Missing (%)** |
| --- | --- | --- |
| gender | 13 | 2.7 |
| age | 23 | 4.8 |
| HT | 37 | 7.6 |
| HTY | 37 | 7.6 |
| DM | 41 | 8.5 |
| DMY | 41 | 8.5 |
| Smoking | 39 | 8.1 |
| LDL_C | 58 | 12 |
| HDL_C | 58 | 12 |
| TC | 58 | 12 |
| ApoB | 40 | 8.3 |
| CREA | 109 | 22.5 |
| eGFR | 109 | 22.5 |

**Supplementary Table 2** Alternative PWV averaging using unilateral maximum value instead of bilateral mean

| **Characteristic** | **OR** | **Std. Error** | **z** | ***P*** | **95% CI low** | **95% CI high** |
| --- | --- | --- | --- | --- | --- | --- |
| Intercept | 0.00 | 2.000 | -3.33 | <0.001 | 0.00 | 0.06 |
| maxPWV | 1.09 | 0.017 | 4.88 | <0.001 | 1.05 | 1.13 |
| TyG | 1.83 | 0.207 | 2.90 | 0.004 | 1.22 | 2.76 |
| genderM | 2.23 | 0.275 | 2.93 | 0.003 | 1.32 | 3.87 |
| eGFR | 0.99 | 0.006 | -2.03 | 0.042 | 0.97 | 1.00 |
| °Carotid PWV reference value was defined as the mean of left and right sides, with a normal cut-off of ≤21.56 m/s (derived from the normal reference values of left ≤21.00 m/s and right ≤22.12 m/s, as specified in cerebrovascular function test reports).  Abbreviations: LAA, large-artery atherosclerosis; SVO, small-vessel occlusion; PWV, pulse wave velocity; TyG, triglyceride–glucose index; eGFR, estimated glomerular filtration rate; OR, odds ratio; CI, confidence interval. | | | | | | |

**Supplementary Table 3** Including patients with severe hepatic or renal dysfunction in the model

| **Characteristic** | **OR** | **Std. Error** | **z** | ***P*** | **95% CI low** | **95% CI high** |
| --- | --- | --- | --- | --- | --- | --- |
| Intercept | 0.01 | 1.700 | -2.73 | 0.006 | 0.00 | 0.26 |
| pwv | 1.06 | 0.017 | 3.30 | <0.001 | 1.02 | 1.09 |
| TyG | 1.55 | 0.170 | 2.58 | 0.010 | 1.12 | 2.18 |
| genderM | 1.95 | 0.257 | 2.60 | 0.009 | 1.19 | 3.26 |
| eGFR | 0.99 | 0.006 | -1.78 | 0.075 | 0.98 | 1.00 |
| °Carotid PWV reference value was defined as the mean of left and right sides, with a normal cut-off of ≤21.56 m/s (derived from the normal reference values of left ≤21.00 m/s and right ≤22.12 m/s, as specified in cerebrovascular function test reports).  Abbreviations: LAA, large-artery atherosclerosis; SVO, small-vessel occlusion; PWV, pulse wave velocity; TyG, triglyceride–glucose index; eGFR, estimated glomerular filtration rate; OR, odds ratio; CI, confidence interval. | | | | | | |

**Supplementary Table 4** Multiple-imputation sensitivity analysis

| **Characteristic** | **pooled** **OR** | **Std. Error** | **z** | ***P*** | **95% CI low** | **95% CI high** |
| --- | --- | --- | --- | --- | --- | --- |
| Intercept | 0.00 | 1.522 | -3.85 | <0.001 | 0.00 | 0.06 |
| pwv | 1.05 | 0.014 | 3.86 | <0.001 | 1.03 | 1.08 |
| TyG | 1.84 | 0.161 | 3.77 | <0.001 | 1.34 | 2.52 |
| genderM | 2.00 | 0.210 | 3.33 | 0.001 | 1.33 | 3.03 |
| eGFR | 0.99 | 0.006 | -1.18 | 0.238 | 0.98 | 1.00 |
| °Carotid PWV reference value was defined as the mean of left and right sides, with a normal cut-off of ≤21.56 m/s (derived from the normal reference values of left ≤21.00 m/s and right ≤22.12 m/s, as specified in cerebrovascular function test reports).  Abbreviations: LAA, large-artery atherosclerosis; SVO, small-vessel occlusion; PWV, pulse wave velocity; TyG, triglyceride–glucose index; eGFR, estimated glomerular filtration rate; OR, odds ratio; CI, confidence interval. | | | | | | |

**Supplementary Table 5. Clinical-model augmentation and LASSO robustness analyses**

| **Variables** | **AUC** | **95% CI** | **Sensitivity** | **Specificity** | **Youden index** | **ΔAUC (reference − model)** | **P (paired DeLong vs reference)** | **P (AUC>0.5)** |
| --- | --- | --- | --- | --- | --- | --- | --- | --- |
| Clinical model | 0.636 | 0.572–0.700 | 0.445 | 0.771 | 0.216 | 0.055 | 0.020 | <0.001 |
| Clinical + PWV + TyG | 0.690 | 0.629–0.752 | 0.420 | 0.860 | 0.281 | Ref | - | <0.001 |
| LASSO-selected refit | 0.685 | 0.623–0.746 | 0.655 | 0.654 | 0.309 | 0.006 | 0.842 | <0.001 |
| **Notes:** Reference model is the prespecified clinical model augmented with PWV and TyG. AUCs and 95% CIs were estimated using DeLong’s method. Sensitivity and specificity are reported at the Youden-optimal threshold. Paired DeLong tests compare each model against the reference. The LASSO-selected refit model includes DMY, HDL-C, PWV, and TyG (selected at lambda.1se and refitted using standard logistic regression). | | | | | | | | |
