# Supplementary figures and images for: Combined pulse wave velocity and triglyceride–glucose index to discriminate large-artery atherosclerosis from small-vessel occlusion in acute ischaemic stroke: a single-centre retrospective study
Source: Front Neurol. 2026 Mar 31;17:1775302. doi: 10.3389/fneur.2026.1775302 (PMC13076111; doi:10.3389/fneur.2026.1775302)

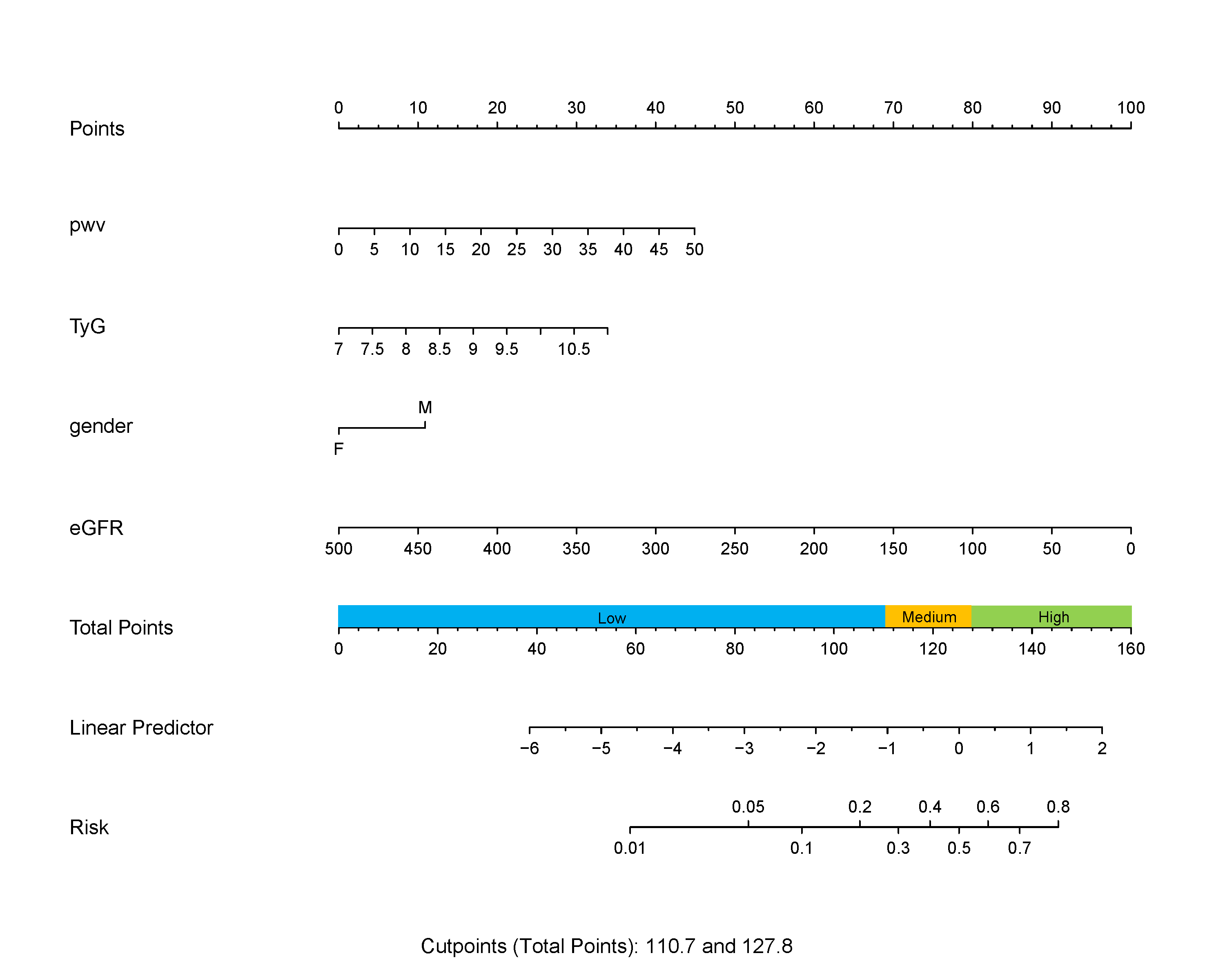

Supplement: Supplementary file 3 [file Image_2.tif]
